# Methodological Issues in Using a Common Data Model of COVID-19 Vaccine Uptake and Important Adverse Events of Interest: Feasibility Study of Data and Connectivity COVID-19 Vaccines Pharmacovigilance in the United Kingdom

**DOI:** 10.2196/37821

**Published:** 2022-08-22

**Authors:** Gayathri Delanerolle, Robert Williams, Ana Stipancic, Rachel Byford, Anna Forbes, Ruby S M Tsang, Sneha N Anand, Declan Bradley, Siobhán Murphy, Ashley Akbari, Stuart Bedston, Ronan A Lyons, Rhiannon Owen, Fatemeh Torabi, Jillian Beggs, Antony Chuter, Dominique Balharry, Mark Joy, Aziz Sheikh, F D Richard Hobbs, Simon de Lusignan

**Affiliations:** 1 Nuffield Department of Primary Care Health Sciences University of Oxford Oxford United Kingdom; 2 Royal College of General Practitioners London United Kingdom; 3 Centre for Public Health Queen’s University Belfast Belfast United Kingdom; 4 Public Health Agency Belfast United Kingdom; 5 Population Data Science Swansea University Swansea United Kingdom; 6 Usher Institute University of Edinburgh Edingburgh United Kingdom

**Keywords:** Systematized Nomenclature of Medicine, COVID-19 vaccines, COVID-19, sinus thrombosis, anaphylaxis, pharmacovigilance, vaccine uptake, medical outcome, clinical coding system, health database, health information, clinical outcome, vaccine effect, data model

## Abstract

**Background:**

The Data and Connectivity COVID-19 Vaccines Pharmacovigilance (DaC-VaP) UK-wide collaboration was created to monitor vaccine uptake and effectiveness and provide pharmacovigilance using routine clinical and administrative data. To monitor these, pooled analyses may be needed. However, variation in terminologies present a barrier as England uses the Systematized Nomenclature of Medicine Clinical Terms (SNOMED CT), while the rest of the United Kingdom uses the Read v2 terminology in primary care. The availability of data sources is not uniform across the United Kingdom.

**Objective:**

This study aims to use the concept mappings in the Observational Medical Outcomes Partnership (OMOP) common data model (CDM) to identify common concepts recorded and to report these in a repeated cross-sectional study. We planned to do this for vaccine coverage and 2 adverse events of interest (AEIs), cerebral venous sinus thrombosis (CVST) and anaphylaxis. We identified concept mappings to SNOMED CT, Read v2, the World Health Organization’s *International Classification of Disease Tenth Revision* (ICD-10) terminology, and the UK Dictionary of Medicines and Devices (dm+d).

**Methods:**

Exposures and outcomes of interest to DaC-VaP for pharmacovigilance studies were selected. Mappings of these variables to different terminologies used across the United Kingdom’s devolved nations’ health services were identified from the Observational Health Data Sciences and Informatics (OHDSI) Automated Terminology Harmonization, Extraction, and Normalization for Analytics (ATHENA) online browser. Lead analysts from each nation then confirmed or added to the mappings identified. These mappings were then used to report AEIs in a common format. We reported rates for windows of 0-2 and 3-28 days postvaccine every 28 days.

**Results:**

We listed the mappings between Read v2, SNOMED CT, ICD-10, and dm+d. For vaccine exposure, we found clear mapping from OMOP to our clinical terminologies, though dm+d had codes not listed by OMOP at the time of searching. We found a list of CVST and anaphylaxis codes. For CVST, we had to use a broader cerebral venous thrombosis conceptual approach to include Read v2. We identified 56 SNOMED CT codes, of which we selected 47 (84%), and 15 Read v2 codes. For anaphylaxis, our refined search identified 60 SNOMED CT codes and 9 Read v2 codes, of which we selected 10 (17%) and 4 (44%), respectively, to include in our repeated cross-sectional studies.

**Conclusions:**

This approach enables the use of mappings to different terminologies within the OMOP CDM without the need to catalogue an entire database. However, Read v2 has less granular concepts than some terminologies, such as SNOMED CT. Additionally, the OMOP CDM cannot compensate for limitations in the clinical coding system. Neither Read v2 nor ICD-10 is sufficiently granular to enable CVST to be specifically flagged. Hence, any pooled analysis will have to be at the less specific level of cerebrovascular venous thrombosis. Overall, the mappings within this CDM are useful, and our method could be used for rapid collaborations where there are only a limited number of concepts to pool.

## Introduction

COVID-19 vaccination is the best option for controlling the current pandemic, with data about uptake and pharmacovigilance (the science and activities relating to the detection, assessment, understanding, and prevention of any side effects of a vaccine or drug) therefore essential for monitoring its progress. Since COVID-19 was first identified in Wuhan, China, at the end of 2019, the virus has spread worldwide, with more than 190 million confirmed cases and over 5.9 million COVID-19–related deaths as of Feburary 28, 2022 [1,2]. Worldwide, most health care systems have opted for a vaccination strategy to protect public health by reducing the incidence but, most importantly, serious outcomes leading to hospitalization and death. Five vaccines have been approved for use in the United Kingdom by the Medicines and Healthcare products Regulatory Agency (MHRA). The first 2, Pfizer-BioNTech and Oxford-AstraZeneca, have been used since December 2020 and January 2021, respectively [3,4]. Real-world data (RWD) suggest that these vaccines are effective [5,6] in preventing severe disease and death; RWD in medicine are data derived from any number of sources that are associated with outcomes in a heterogeneous patient population in real-world settings, such as patient surveys, clinical trials, and observational cohort studies. However, there has been concern about the risk of adverse effects, such as thrombotic thrombocytopenia and anaphylaxis [7,8]. It is important to be able to monitor these at scale to give power to detect potential associations with rare adverse events of interest (AEIs). AEIs are medical conditions arising after the administration of a vaccine, not necessarily causally linked.

Medical record systems enable information flow beyond organizational boundaries. General practices with their own information technology (IT) systems record millions of patient interactions daily. A challenge for this partnership is the heterogeneity of routine primary care data due to variation in the clinical terminologies used across the 4 UK nations. The Data and Connectivity COVID-19 Vaccines Pharmacovigilance (DaC-VaP) collaboration was formed to explore vaccine effectiveness, uptake, and safety across the United Kingdom. England has transitioned to the Systematized Nomenclature of Medicine Clinical Terms (SNOMED CT) and has not updated Read v2 since April 2016 [9]. Read v2 is a clinical terminology system (5-byte version) that was developed by Dr. James Read. However, the devolved nations (Scotland, Wales, and Northern Ireland) still use the Read terminology. In addition, the levels of granularity and hierarchy of the 2 are incompatible, making comparison of the results of any analysis a challenge. Primary care data have not been included in the Northern Ireland component of the study because systems to make anonymized primary care data available for research are under development.

The use of a common data model (CDM) could provide a solution faced by many looking to aggregate data from different sources [10,11]. CDMs use logic and semantics (in the case of the OMOP CDM, having 1 standard reference vocabulary per domain so that everyone is “speaking the same language”) to standardize data and enable data from different sources to be used in pooled analyses. The use of CDMs is common within clinical research, and at present, 3 are cited in many studies: (1) the Observational Medical Outcomes Partnership CDM (OMOP; used to be a partnership project but now only designates a type of a CDM for RWD/evidence in clinical research), (2) the US Food and Drug Administration (FDA) Sentinel CDM (the Sentinel Operations Center [SOC] coordinates the network of Sentinel data partners and leads the development of the Sentinel common data model [SCDM], a standard data structure that allows the data partners to quickly execute distributed programs against local data), and (3) the Patient-Centered Outcomes Research Institute (PCORI) CDM (PCORnet; facilitates the sharing of information across PCORI's wider network and is based on the mini-SCDM) [12]. The OMOP CDM, the most cited of the 3, enables the transformation of data from diverse observational databases into a common format using a standardized vocabulary [13]. The OMOP CDM includes different data domains required for observational studies, including demographics, vaccine exposure, and AEIs relevant to this study [14].

Other groups, including the National COVID Cohort Collaborative (N3C) in the United States, have faced challenges in how to achieve harmonization between data sources. Although this was customized and drew together data from different sources and CDMs, the N3C also extensively used OMOP [15]. We carried out this study, therefore, to test the feasibility of using the OMOP CDM for comparisons of vaccine uptake and AEIs across the 4 UK nations.

Our primary aim is to assess the feasibility of using the OMOP CDM to report the incidence of exemplar AEIs following COVID-19 vaccination across the DaC-VaP collaboration and report these as repeated cross-sectional analyses. The objectives of the study include the following:

- To test the validity of the mappings within the OMOP Automated Terminology Harmonization, Extraction, and Normalization for Analytics (ATHENA) online browser to our exemplar AEIs. ATHENA is a repository of all the latest OMOP CDM vocabularies and mappings are hosted and can be searched and downloaded.
- To report the vaccine uptake rate across the United Kingdom, stratified by age group, sex, vaccine type, and ethnicity, with the goal of reporting a UK-wide vaccination uptake rate. We differentiated people who have had their first and second doses.
- To report the rates for England, Scotland, Wales, and Northern Ireland and overall for the 2 exemplar AEIs, cerebral venous sinus thrombosis (CVST) and anaphylaxis.

## Methods

### Overview

We used the OMOP ATHENA online browser to identify mappings to SNOMED CT, Read v2 terminology, and the *International Classification of Disease Tenth Revision* (ICD-10). We reported vaccine exposure using SNOMED CT for vaccine administration and *Dictionary of Medicines and Devices* (dm+d; National Health Service [NHS]) codes for vaccine prescriptions; dm+d is a dictionary of descriptions and codes which represent medicines and devices in use across the NHS. Each national team validated these mappings and any differences between the terms they would use to represent each concept discussed with a decision made by consensus. We then used to create monthly reports of vaccine coverage and AEIs. We elected to use CVST and anaphylaxis as demonstration AEIs [16].

### Settings

The data were drawn from the data sources of the 4 DaC-VaP partners.

#### English Data

The data from England are from the Oxford Royal College of General Practitioners (RCGP) Research and Surveillance Centre (RSC), 1 of Europe’s oldest surveillance networks. It is now in its 53rd season of operation, working alongside Public Health England (PHE) [17]. The RSC has been involved in monitoring studies of influenza vaccine safety. This network is active in COVID-19 research, including the PRINCIPLE (Platform Randomized Trial of Treatments in the Community for Epidemic and Pandemic Illnesses) trial [18]. The RSC is the surveillance platform of the Oxford RCGP Clinical Informatics Digital Hub (ORCHID, a secure data processing environment) [19,20].

#### Scottish Data

The EAVE II data of 5.4 million people registered in general practices in Scotland track COVID-19 within the Scottish population. This effort has led to impactful findings used by the Scottish and UK governments to respond to the COVID-19 pandemic.

#### Welsh Data

The Secure Anonymised Information Linkage (SAIL) databank is a trusted research environment (TRE), a Wales-wide research resource focused on improving health, well-being, and services, which includes primary care general practitioner (GP) records, secondary care hospital data, and emergency services data, along with a range of administrative, governmental, education, social care, and specialist audit, register, and services data. These data are also used by the National Institute for Health and Care Excellence (NICE) to shape policies that cover England and Wales. SAIL is powered by the Secure e-Research Platform (SeRP, a technology platform and service that enables the SAIL databank and other TREs and platforms in the United Kinngdom and worldwide).

#### Northern Ireland Data

Data are accessed through the Business Services Organisation Honest Broker Service (HBS), which also uses SeRP. It has available GP registration (but not primary care clinical records), the enhanced prescribing database, emergency department attendances, hospital admissions, COVID-19 testing, and the vaccine management system.

### Study Design

We performed repeated cross-sectional reports of the incidence of the AEIs in the vaccinated population in a single time interval postvaccination.

#### Phase 1: Validating OMOP Mapping and Creating Searches for Distributed Analyses

We searched the OMOP CDM for the concepts of interest using the ATHENA online browser. These concepts were demographic details, COVID-19 vaccine uptake, and the AEIs. The demographic and socioeconomic status (SES) data of interest were age and sex, and the SES was divided into quintiles (quintile 5 being the most deprived). We also collected data on obesity (defined as the latest BMI≥30 or coded as obesity; the BMI is a measure that uses your height and weight to work out whether your weight is healthy) and smoking status (current, ex-, or never smoked).

We compared the linkages flagged by the OMOP ATHENA online browser with those currently used across the 4 nations. We reported any differences and achieved a consensus as to which terms/codes will be used in each nation.

OMOP also maps to the *Medical Dictionary for Regulatory Activities* (MedDRA), and if the method in this protocol became established, considerations for enabling reporting of pharmacovigilance study findings mapped to MedDRA.

Each DaC-VaP partner nation will restrict its ATHENA search using the VOCAB (vocabulary) tool to SNOMED CT or Read v2, as relevant, and dm+d.

The medication dictionary dm+d is made up of a hierarchy of generic terms (termed “virtual”) and real prescribable items (termed “actual”). The dm+d use case for the COVID-19 vaccine is set out below:

- Virtual therapeutic moiety (VTM): top of the hierarchy (type of therapy indicator, eg, COVID-19 vaccine); in this use case, this is the COVID-19 vaccine.
- Virtual medicinal product (VMP): This is the next level of notional product and allows vaccine types (COVID-19 vaccine type: mRNA or vector); in our use case, messenger RNA (mRNA) vaccines and their manufacturers were to be distinguished from recombinant vaccines.
- Virtual medicinal product pack (VMPP): This is the notional product pack for the medical VMP, for example, a 6-dose multidose vial.
- Actual medicinal products (AMPs): These are the medicinal products prescribed to an individual; for example, vaccine brands (Pfizer-BioNTech, Moderna).
- Actual medicinal product packs (AMPPs): These are the distribution packs of medicinal products.

The analyst team from each nation reported whether they included all the terms identified from their search of OMOP for mapping to their terminology using ATHENA and whether they added others they routinely use.

#### Phase 2: Monthly Reports and Aggregation of Results

We ran these searches monthly to produce a monthly output of vaccine coverage by demographic group and reported the incidence of our AEIs.

Vaccine uptake was reported as the percentage of adults vaccinated per nation and stratified by age, sex, smoking status, and obesity. We reported 2 exemplars of AEIs following vaccination using 2 time windows (0-2 and 3-28 days).

### Cross Sections, Exposures, and Outcomes

DaC-VaP partners ran cross-sectional studies for the previous 28 days.

The first search started on December 8, 2020 (first dose of the Pfizer vaccine given in the United Kingdom), and the second search on January 5, 2021. These ran in 28-day intervals (February 2, March 2, March 30, April 27-August 17, 2021).

The cross sections included all individuals registered with general practices on the date of vaccination and remained registered for 28 days. The outputs were reported in the following age bands: <16 years old, 16-39 years, 40-64 years, and 65 years and older. Mortality in the postvaccination period were also reported for those with AEIs.

We reported by vaccine brand, including reporting unknown vaccines. We presumed that the unknown vaccine brand for December 2020 was Pfizer-BioNTech, as Oxford-AstraZeneca was unavailable until January 2021 and other vaccine types later.

We aimed to include statistical reporting and disproportional analysis metrics, a proportional reporting ratio (PRR), and a reporting odds ratio (ROR). We used a Bayesian method that provided a framework to combine prior information/knowledge and data to account for conceptual transparency. Our aim was to use IC = log_2_ (observed + 1/2)/(expected + 1/2).

### Ethical Considerations

The DaC-VaP collaborators had individual ethical control of their data. No data were reported that might risk identifying individuals. Where less than 5 individuals were in a group, this was reported as <5. This study aims to demonstrate the potential of the DaC-VaP collaboration to report outcomes of interest.

#### English Data

The University of Oxford complies with the General Data Protection Regulation and the NHS Digital Data Security and Protection Policy [21]. This study was approved by the Health Research Authority Research Ethics Committee (21/HRA/2786; (Integrated Research Application ID 301740). ORCHID meets the NHS Digital Data Security and Protection Toolkit requirements. [22].

#### Scottish Data

Ethical permission for this study was granted by the South-East Scotland Research Ethics Committee (#314 02; 12/SS/0201). The Public Benefit and Privacy Panel Committee of Public Health Scotland (#315) approved the linkage and analysis of the de-identified data sets for this project (#1920-0279).

#### Welsh Data

This study made use of anonymized data held in the SAIL databank. We used data provided by patients and collected by the NHS as part of their care and support. All research conducted was completed after the permission and approval of the SAIL independent Information Governance Review Panel (IGRP; project number 0911).

#### Northern Ireland Data

Nothern Irish data were accessed from the Business Services Organization HBS, which provided de-identified linked data via SeRP. All research conducted was approved by the HBS Governance Board (HBSGB; project number 064).

## Results

### Study Concepts Within the OMOP CDM

We initially reported whether the data items or clinical concepts required for the study existed within the OMOP CDM and whether there were mappings to SNOMED CT, Read v2, or dm+d (Table 1).

We then reported the components by terminology (Table 2). Age did not map to the Read v2 terminology, but this is of no practical significance. We noted that the SES only exists as a generic concept in OMOP and that a custom mapping would be required. There was no mapping of vaccine dose (first or second) to Read v2. However, like age, this would not be practically important as these data are well ordered in the DaC-VaP collaborators’ data sources.

Of most importance are the AEIs. For CVST, the specific concept exists within OMOP, and it also exists in SNOMED CT: SNOMED concept IDs 95455008 and19522900 from CVST (concept ID 195229008). For anaphylaxis, SNOMED CT and the ATHENA online browser showed 130 and 161 items, respectively. SNOMED CT and OMOP had 16 and 15 items, respectively. Read v2 codes were generic for CVST and anaphylaxis. Of these, those relevant to vaccination are shown in Tables 2, 3, 4, and 5 for England, Wales, Scotland, and Nothern Ireland, respectively.

**Table 1:** Variables included in the CDM^a^ conceptual mapping exercise, with counts subsequently reported monthly.

Data item			OMOP^b^ (Y=yes, N=no)		Data source
**Demographics**					
	Gender	Y		Standardized sex (gender) codes are used in OMOP CDM mapping. Date of birth and age concepts also exist.	
	Age band	Y		Date of birth and age concepts exist in OMOP.	
**SES^c^ quintile**					
	The Index of Multiple Deprivation (IMD, a set of relative measures of deprivation for small areas [lower-layer super-output areas) across England, based on 7 domains of deprivation), in England, the Scottish Index of Multiple Deprivation (SIMD), the Welsh Index of Multiple Deprivation (WIMD), and the Northern Ireland Multiple Deprivation Measure (NIMDM)	N		Does not exist in the OMOP CDM. It can be introduced as a custom mapping in all UK databases within OMOP. It is to be harmonized across the DaC-VaP^d^ data partners (quintile 5 most deprived, quintile 1 least deprived).	
**Other characteristics**					
	BMI>30/obesity	Y		Will be found in the measurement table or from a diagnosis of obesity.	
	Smoking status	Y		Will be found in the observation table.	
**Vaccination**					
	Vaccine type	Y		Will be found in the drug, procedure, and event tables. For England, the source codes are dm+d^e^ or SNOMED CT^f^.	
	Vaccine dose	Y		In vaccine administration.	
	Vaccination date	Y		Date of event when the event is COVID-19 vaccination.	
**Exemplar AEIs^g^**					
	CVST^h^	Y		Will be found in the condition table. It is mapped to SNOMED CT or Read v2. We did not include medications in this feasibility study.	
	Anaphylaxis	Y		Will be found in the condition table. It is mapped to SNOMED CT or Read v2. We did not include medications in this feasibility study.	

^a^CDM: common data model.

^b^OMOP: Observational Medical Outcomes Partnership.

^c^SES: socioeconomic status.

^d^DaC-VaP: Data and Connectivity COVID-19 Vaccines Pharmacovigila.

^e^dm+d: Dictionary of Medicines and Devices.

^f^SNOMED CT: Systematized Nomenclature of Medicine Clinical Trials.

^g^AEI: adverse event of interest.

^h^CVST: cerebral venous sinus thrombosis.

**Table 2:** Study concepts identified within OMOP^a^ and ICD-10^b^, and any mapping to SNOMED CT^c^, Read v2, and dm+d^d^.

Primary term	OMOP ATHENA^e^ concept ID	ICD-10	dm+d	SNOMED CT	Read v2
CVST^f^ (nonstandard to standard map)	10083037	I63.6, I67.6, U07.7 (vaccine caused adverse effects) and P3.344 (CVST in hospitalized adults)	N/A^g^	4102202	N/A
CVST (standard to nonstandard map)	10083037	I63.6, 167.6, UO7.7 (vaccine caused adverse effects)	N/A	4102202	N/A
Anaphylaxis (localized)	4034658	T78.2 (anaphylactic shock unspecified)	N/A	40316757 (systemic), 42536383 (anaphylactic shock), 4294049 (sudden onset), 2084167 (allergic), 4084167 (acute allergic reaction), 441202 (nonstandard to standard OMOP map), 441202, 40640468 (generalized)	N/A
Anaphylaxis	441202	45537000 (anaphylactic shock unspecified)	N/A	40316757 (systemic), 40640468 (generalized)	N/A
Anaphylaxis (anaphylactic shock due to adverse effect of correct medicinal substance properly administered)	45376003	45537000 (anaphylactic shock unspecified), 19746	N/A	4254051 (drug or medicament), 441297 (adverse reaction to drug)	N/A
Anaphylaxis (drug induced)	241937000	45537000 (anaphylactic shock unspecified)	N/A	46274027, 4084168 (nonstandard OMOP)	N/A
Anaphylaxis (procedure)	42537947	45537000 (anaphylactic shock unspecified)	N/A	44807057 (anaphylaxis care), 4021200 (care of patient states), 42537947 (nonstandard to standard OMOP map), 44807057 (standard to nonstandard OMOP map)	N/A
Anaphylaxis (due to substance)	4221182	45537000 (anaphylactic shock unspecified)	N/A	4022675 (substance), 4294049 (sudden onset), 441202 (anaphylaxis), 4221182 (nonstandard to standard OMOP map), 4083868 (standard to nonstandard OMOP map)	N/A

^a^OMOP: Observational Medical Outcomes Partnership.

^b^ICD-10: *International Classification of Disease Tenth Revision*.

^c^SNOMED CT: Systematized Nomenclature of Medicine Clinical Trials.

^d^dm+d: Dictionary of Medicines and Devices.

^e^ATHENA: Automated Terminology Harmonization, Extraction, and Normalization for Analytics.

^f^CVST: cerebral venous sinus thrombosis.

^g^N/A: not applicable.

**Table 3:** Study concepts identified within OMOP^a^ and any mapping to ICD-10^b^, dm+d^c^, SNOMED CT^d^, or Read v2 in Scotland.

Primary term	OMOP ATHENA^e^ concept ID	ICD-10	dm+d	SNOMED CT	Read v2
CVST^f^ (nonstandard to standard map)	10083037	I63.6, 167.6, UO7.7 (vaccine caused adverse effects) and P3.344 (CVST in hospitalized adults)	N/A^g^	N/A	N/A
CVST (standard to nonstandard map)	10083037	I63.6, 167.6, UO7.7 (vaccine caused adverse effects)	N/A	N/A	N/A
Cerebral vein thrombosis	45446702	I63.6, 167.6, UO7.7 (vaccine caused adverse effects)	N/A	N/A	G67A
Thrombosis of central nervous system venous sinus NOS	3534267	I63.6, 167.6, UO7.7 (vaccine caused adverse effects)	N/A	N/A	F051z
Thrombophlebitis of central nervous system venous sinuses	4100223	I63.6, 167.6, UO7.7 (vaccine caused adverse effects)	N/A	N/A	F053
Nonpyogenic venous sinus thrombosis	45456755	I63.6, 167.6, UO7.7 (vaccine caused adverse effects)	N/A	N/A	G676
Anaphylaxis (localized)	4034658	45537000 (anaphylactic shock unspecified)	N/A	N/A	N/A
Anaphylaxis	441202	45537000 (anaphylactic shock unspecified)	N/A	N/A	N/A
Anaphylaxis (anaphylactic shock due to adverse effect of correct medicinal substance properly administered)	45376003	45537000 (anaphylactic shock unspecified), 19746	N/A	N/A	N/A
Anaphylaxis (drug induced)	241937000	45537000 (anaphylactic shock unspecified)	N/A	N/A	N/A
Anaphylaxis (procedure)	42537947	45537000 (anaphylactic shock unspecified)	N/A	N/A	N/A
Anaphylaxis (due to substance)	4221182	45537000 (anaphylactic shock unspecified)	N/A	N/A	N/A

^a^OMOP: Observational Medical Outcomes Partnership.

^b^ICD-10: *International Classification of Disease Tenth Revision*.

^c^dm+d: *Dictionary of Medicines and Devices*.

^d^SNOMED CT: Systematized Nomenclature of Medicine Clinical Trials.

^e^ATHENA: Automated Terminology Harmonization, Extraction, and Normalization for Analytics.

^f^CVST: cerebral venous sinus thrombosis.

^g^N/A: not applicable.

**Table 4:** Study concepts identified within OMOP^a^ and any mapping to ICD-10^b^, dm+d^c^, SNOMED CT^d^, or Read v2 in Wales.

Primary term	OMOP ATHENA^e^ concept ID	ICD-10	dm+d	SNOMED CT	Read v2
CVST^f^ (nonstandard to standard map)	10083037	I63.6, I67.6, U07.7 (vaccine caused adverse effects) and P3.344 (CVST in hospitalized adults)	N/A^g^	N/A	N/A
CVST (standard to nonstandard map)	10083037	I63.6, 167.6, UO7.7 (vaccine caused adverse effects)	N/A	N/A	N/A
Anaphylaxis (localized)	4034658	T78.2 (anaphylactic shock unspecified)	N/A	N/A	N/A
Anaphylaxis	441202	45537000 (anaphylactic shock unspecified)	N/A	N/A	SN50.11
Anaphylaxis (anaphylactic shock due to adverse effect of correct medicinal substance properly administered)	45376003	45537000 (anaphylactic shock unspecified), 19746	N/A	N/A	SN50110
Anaphylaxis (drug induced)	241937000	45537000 (anaphylactic shock unspecified)	N/A	N/A	SN50.00, 14M5.00
Anaphylaxis (procedure)	42537947	45537000 (anaphylactic shock unspecified)	N/A	N/A	SN50.11, SN50.00, 14M5.00
Anaphylaxis (due to substance)	4221182	45537000 (anaphylactic shock unspecified)	N/A	N/A	SN50.11, SN50.00, 14M5.00

^a^OMOP: Observational Medical Outcomes Partnership.

^b^ICD-10: *International Classification of Disease Tenth Revision*.

^c^dm+d: *Dictionary of Medicines and Devices*.

^d^SNOMED CT: Systematized Nomenclature of Medicine Clinical Trials.

^e^ATHENA: Automated Terminology Harmonization, Extraction, and Normalization for Analytics.

^f^CVST: cerebral venous sinus thrombosis.

^g^N/A: not applicable.

**Table 5:** Study concepts identified within OMOP^a^ and any mapping to ICD-10^b^, dm+d^c^, SNOMED CT^d^, or Read v2 in Northern Ireland.

Primary term	OMOP ATHENA^e^ concept ID	ICD-10	dm+d	SNOMED CT	Read v2
CVST^f^ (nonstandard to standard map)	10083037	I63.6, 167.6, UO7.7 (vaccine caused adverse effects) and P3.344 (CVST in hospitalized adults)	N/A^g^	N/A	N/A
CVST (standard to nonstandard map)	10083037	I63.6, 167.6, UO7.7 (vaccine caused adverse effects)	N/A	N/A	N/A
Anaphylaxis (localized)	4034658	45537000 (anaphylactic shock unspecified)	N/A	N/A	N/A
Anaphylaxis	441202	45537000 (anaphylactic shock unspecified)	N/A	N/A	N/A
Anaphylaxis (anaphylactic shock due to adverse effect of correct medicinal substance properly administered)	45376003	45537000 (anaphylactic shock unspecified), 19746	N/A	N/A	N/A
Anaphylaxis (drug induced)	241937000	45537000 (anaphylactic shock unspecified)	N/A	N/A	N/A
Anaphylaxis (procedure)	42537947	45537000 (anaphylactic shock unspecified)	N/A	N/A	N/A
Anaphylaxis (due to substance)	4221182	45537000 (anaphylactic shock unspecified)	N/A	N/A	N/A

^a^OMOP: Observational Medical Outcomes Partnership.

^b^ICD-10: *International Classification of Disease Tenth Revision*.

^c^dm+d: *Dictionary of Medicines and Devices*.

^d^SNOMED CT: Systematized Nomenclature of Medicine Clinical Trials.

^e^ATHENA: Automated Terminology Harmonization, Extraction, and Normalization for Analytics.

^f^CVST: cerebral venous sinus thrombosis.

^g^N/A: not applicable.

### Vaccine Exposure

COVID-19 vaccine exposure was well recorded with dm+d and SNOMED CT. The vaccine unsurprisingly was listed as a VTM at the top of the drug dictionary hierarchy, with VMPs created for each vaccine type. There were virtual and actual packs and products to match the vaccines available. Additional administration and vaccine-type clinical terms were also within SNOMED CT (Table 6). Finally, we found a small number of vaccine administration codes within dm+d that were not mapped to OMOP.

**Table 6:** COVID-19 vaccine concepts.

Vaccine brand/generic/administration	Administration, n	Number of dm+d^a^ or SNOMED CT^b^ codes, n						Ingredients, n
		VTM^c^	VMP^d^	VMPP^e^	AMPP^f^	AMP^g^		
Generic COVID-19	N/A^h^	1	N/A	N/A	N/A	N/A	N/A	
Generic mRNA^i^	3	N/A	N/A	N/A	N/A	N/A	N/A	
Generic recombinant	N/A	N/A	N/A	N/A	N/A	N/A	3	
Vaccine administration	1	N/A	N/A	N/A	N/A	N/A	N/A	
COVID-19 vaccine administration	6	N/A	N/A	N/A	N/A	N/A	N/A	
COVID-19 1st dose vaccine administration	2	N/A	N/A	N/A	N/A	N/A	N/A	
COVID-19 2nd dose vaccine administration	2	N/A	N/A	N/A	N/A	N/A	N/A	
Oxford-AstraZeneca(	N/A	N/A	1	4	4	1	N/A	
Moderna	N/A	N/A	1	2	2	1	N/A	
Pfizer-BioNTech	N/A	N/A	1	2	2	1	N/A	

^a^dm+d: Dictionary of Medicines and Devices.

^b^SNOMED CT: Systematized Nomenclature of Medicine Clinical Trials.

^c^VTM: virtual therapeutic moiety.

^d^VMP: virtual medicinal product.

^e^VMPP: virtual medicinal product pack.

^f^AMPP: actual medicinal product pack.

^g^AMP: actual medicinal product.

^h^N/A: not applicable.

## Discussion

### Principle Findings

This study shows a mapping method to identify codes relevant to CVST and anaphylaxis using the OMOP CDM to link common concepts required for COVID-19 vaccine pharmacovigilance to different terminologies relevant to the United Kingdom. All our predefined concepts were represented in the OMOP CDM. However, some, such as SES, did not have specific mappings and, thus, custom mappings would need development. We noted that local codes and curation of variables may be used to enable specificity where the concepts are less granular, especially for CVST.

### Comparison With Prior Work

The OMOP CDM may be suboptimal to overcome the limitation in the granularity of the coding systems used for AEIs. As well as being less granular, the Read v2 terminology has not been updated formally since April 2016, so local adaptions have been undertaken in the developed UK nations to enable new conditions and treatments, such as COVID-19 and vaccination, to be recorded.

**Figure 1:**
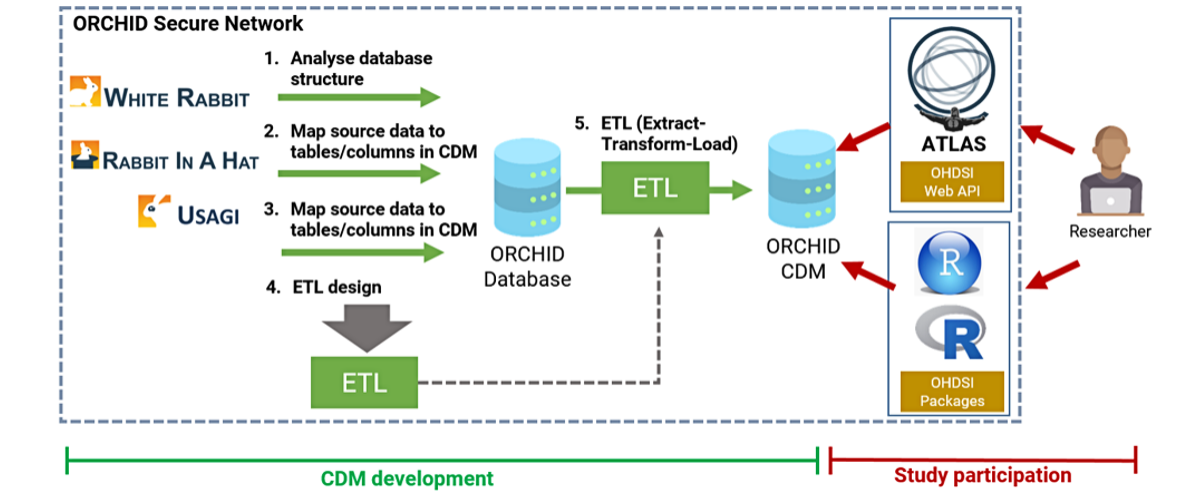
Formal mapping of a database (in this case ORCHID) to the OMOP CDM. We used 3 steps to develop the extract, transform, and load (ETL) design. We tested this specifically in relation to the SQL environment of the ORCHID database. CDM: common data model; OHDSI: Observational Health Data Sciences and Informatics; OMOP: Observational Medical Outcomes Partnership; ORCHID: Oxford RCGP Clinical Informatics Digital Hub.

Conventionally, CDMs, such as OMOP, are used by each database, mapping data and querying them using the script created by 1 of the teams. The cataloguing is carried out using applications such as White Rabbit and Rabbit in a Hat (Figure 1).

### Limitations

The OMOP CDM provides a framework for capturing patient demographic and socioeconomic characteristics, varying vaccine exposure [23], and AEI data. Although others could replicate our approach, avoiding the need to map whole databases, initial findings reflect the relative size of the terminologies we use, and their granularity needs improvement. Therefore, to conduct future research, concepts will require localization to evaluate COVID-19 vaccination associated with CVST and anaphylaxis. We selected dm+d rather than the better-known British National Formulary (BNF), although the latter is mapped to SNOMED CT. Its limitations are that it only lists prescribable drugs. Its chapter headings change from time to time, and it is not mapped to the ATHENA OMOP hierarchy. We considered MedDRA with clincically validated medical terminologies for clinical conditions, medical devices, and medicines that is commonly used to share AEIs mainly with regulators. MeDRA is primaily used to report pharmacovigilance using acute care and clinical trial data. This approach cannot be directly used within primary care but will be considered for future studies.

### Conclusion

Concept mapping to a large number of terminologies, such as within OMOP and its ATHENA online browser, are usable and valuable for those conducting studies that draw together heterogeneous data to perform pooled analyses. Comprehensive mappings have to set a level of granularity that may be more or less specific than the terminologies they map to. Clinical variable curation at a local database level would prove useful to address issues around granularity. This would allow local expert refinement of the mappings that could be used by others looking to do a limited pooled analysis of a small number of clinical concepts. The interconnectivity of the pooled analysis may also support the MHRA’s Sponstaneous Report System used for optimizing patient safety.

## Abbreviations

AEI: adverse event of interest
AMP: actual medicinal product
AMPP: actual medicinal product pack
ATHENA: Automated Terminology Harmonization, Extraction, and Normalization for Analytics
CDM: common data model
CVST: cerebral venous sinus thrombosis
DaC-VaP: Data and Connectivity COVID-19 Vaccines Pharmacovigila
dm+d: Dictionary of Medicines and Devices
FDA: Food and Drug Administration
GP: general practitioner
HBS: Honest Broker Service
IC: *information component*
ICD-10: *International Classification of Disease Tenth Revision*
MedDRA: Medical Dictionary for Regulatory Activities
MHRA: Medicines and Healthcare products Regulatory Agency
N3C: National COVID Cohort Collaborative
NHS: National Health Service
OHDSI: Observational Health Data Sciences and Informatics
OMOP: Observational Medical Outcomes Partnership
ORCHID: Oxford RCGP Clinical Informatics Digital Hub
PCORI: Patient-Centered Outcomes Research Institute
PCORnet: Patient-Centred Outcomes Research Network
RCGP: Royal College of General Practitioners
RSC: Research and Surveillance Centre
RWD: real-world data
SAIL: Secure Anonymised Information Linkage
SCDM: Sentinel common data model
SeRP: Secure e-Research Platform
SES: socioeconomic status
SNOMED CT: Systematized Nomenclature of Medicine Clinical Terms
TRE: trusted research environment
VMP: virtual medicinal product
VMPP: virtual medicinal product pack
VTM: virtual therapeutic moiety
